# Aerobic exercise in adolescents with obesity: preliminary evaluation of a modular training program and the modified shuttle test

**DOI:** 10.1186/1471-2431-7-19

**Published:** 2007-04-19

**Authors:** Peter HC Klijn, Olga H van der Baan-Slootweg, Henk F van Stel

**Affiliations:** 1 Department of Physical Rehabilitation and Therapy, KBCZ, Treatment Center Heideheuvel, Hilversum, The Netherlands; 2 Department of Pediatric Medicine, KBCZ, Treatment Center Heideheuvel, Hilversum, The Netherlands; 3 Department of Medical Decision Making, Leiden University Medical Center, Leiden, The Netherlands; 4 Julius Center for Health Sciences and Primary Care, University Medical Center Utrecht, Utrecht, The Netherlands

## Abstract

**Background:**

Increasing activity levels in adolescents with obesity requires the development of exercise programs that are both attractive to adolescents and easily reproducible. The aim of this study was to develop a modular aerobic training program for adolescents with severe obesity, with a focus on variety, individual targets and acquiring physical skills. We report here the effects on aerobic fitness from a pilot study. Furthermore, we examined the feasibility of the modified shuttle test (MST) as an outcome parameter for aerobic fitness in adolescents with severe obesity.

**Methods:**

Fifteen adolescents from an inpatient body weight management program participated in the aerobic training study (age 14.7 ± 2.1 yrs, body mass index 37.4 ± 3.5). The subjects trained three days per week for 12 weeks, with each session lasting 30–60 minutes. The modular training program consisted of indoor, outdoor and swimming activities. Feasibility of the MST was studied by assessing construct validity, test-retest reliability and sensitivity to change.

**Results:**

Comparing pretraining and end of training period showed large clinically relevant and significant improvements for all aerobic indices: e.g. VO_2 peak _17.5%, effect size (ES) 2.4; W_max _8%, ES 0.8. In addition, a significant improvement was found for the efficiency of the cardiovascular system as assessed by the oxygen pulse (15.8%, ES 1.6).

Construct validity, test-retest reliability and sensitivity to change of the MST were very good. MST was significantly correlated with VO_2 peak _(r = 0.79) and W_max _(r = 0.84) but not with anthropometric measures. The MST walking distance improved significantly by 32.5%, ES 2.5. The attendance rate at the exercise sessions was excellent.

**Conclusion:**

This modular, varied aerobic training program has clinically relevant effects on aerobic performance in adolescents with severe obesity. The added value of our aerobic training program for body weight management programs for adolescents with severe obesity should be studied with a randomized trial. This study further demonstrated that the MST is a reliable, sensitive and easy to administer outcome measure for aerobic fitness in adolescent body weight management trials.

## Background

The prevalence of obesity in children and adolescents is increasing rapidly worldwide [1-3] and this trend is also found in The Netherlands[4].

Along with dietary and behavior treatment components, exercise or physical activity is generally considered one of the cornerstones of pediatric obesity treatment[5]. Increasing energy expenditure may accelerate loss of fat mass by creating a negative energy balance, changes in body composition, and potentiate the maintenance of changes in body composition[6,7].

The majority of studies in overweight or obese children and adolescents have focused on aerobic exercise[6]. These studies indicate that aerobic exercise has little effect on gross measures such as bodyweight and body mass index (BMI), but is usually associated with favorable changes in body composition. Aerobic exercise may decrease body fat, attenuate the loss of lean body mass normally seen during dietary energy restriction and mediate the accumulation of visceral adipose tissue[8]. The latter is associated with cardiovascular risk in the pediatric population[9].

Epstein and colleagues speculated that being able to choose one's own activities may be perceived as having control over being more physically active[7]. Both choice and perceived control has shown to be important determinants of exercise adherence[10]. Obese children and adolescents often lack confidence in their physical ability[11]. Therefore, it is important to balance training guidelines and experimental control over the characteristics of an exercise program versus enhancing self-confidence and perceived self-efficacy[12]. Self-efficacy denotes the belief an individual has in his or her ability to engage in a specific behavior[13]. Thus, physical self-efficacy represents skills in a physical task. The acquisition of skills is needed to promote self-efficacy of adolescents. However, decreasing sedentary activity seems a more promising approach than increasing physical activity for body weight management[14]. Incorporating a variety of physical activities in a structured exercise program possibly represent a method of reinforcing reduction in sedentary behaviours and choosing one's own physical activities under free living conditions.

Resting energy expenditure (REE) is very important because REE represents a significant share of the total energy expenditure[15]. Up to 80% of daily energy expenditure occurs at rest and relative to adipose tissue, lean body mass has a high basal metabolic rate[6]. Hence, preservation of lean body is an effective way of increasing daily energy expenditure and thereby decreasing fat mass. In addition, it may positively influence long-term outcome[7].

Childhood obesity increases the risk of obesity in adulthood where the health risks are well established[16]. Some epidemiological data indicate that aerobic fitness, rather than fatness, may be the most appropriate target for lifestyle interventions [17-23]. Studies in overweight and obese adults have demonstrated that low cardiovascular fitness is as important as BMI for predicting mortality[24,25]. This hypothesis is supported by recent pediatric studies [26-28].

It has been proposed that encouraging the development of physical activity habits in children, and reinforcing these habits in adolescents, helps establish patterns that continue into adulthood[29]. In addition, obesity and low initial levels of fitness may attenuate the positive consequences associated with being active and reduce the motivation to keep up an exercise program[12].

There are several reports in the literature about exercise programs for adolescents with obesity [30-35]. However, the focus of most programs is on long-lasting endurance activities, which in our opinion are boring for the pediatric population. Furthermore, most programs are not easily reproducible due to lack of detail or requirement of special equipment. Therefore, our aim was to develop a modular aerobic exercise program that is attractive to adolescents with obesity and easily reproducible by others. Several requirements can be defined for such an exercise program[36].

First, an exercise program for adolescents should include a variety of physical activities to increase the physical skills of the participants[37]. Second, activities at the beginning of the program should focus on gains in self-confidence and perceived self-efficacy[11]. Third, a training program should be individualized. Fourth, the program should be enjoyable with a wide variation of exercises. Fifth, a program should preferably be developed with an open format.

The second aim of this study is to report on an easily administred exercise test that suits the exercise program. Formal laboratory-based exercise tests with on-line analysis of expired air are considered the gold standard for measuring aerobic training effects[38]. However, these tests are time-consuming, expensive and the required specialized equipment is not widely available[38,39]. In addition, many subjects with obesity find these tests excessively stressful and are reluctant to perform these tests on a routine basis. Therefore, there is a need for a simple practical method of measuring aerobic fitness in adolescents with obesity[40]. For this purpose a test should be incremental and progressive in nature, stressing the subject to a symptom limited maximal performance. Drinkard et al used the 12-minute walk/run in adolescents with obesity[40]. The 12-minute walk/run is a self paced test which may be influenced by motivation. The subject sets the pace throughout the test. This raises the likelihood that maximum cardiorespiratory values are not reached. The incremental, externally paced 20 meter shuttle running test is a widely used field test of functional capacity in children [41-43]. However, the starting speed of the 20 meter shuttle running test is too fast for children with obesity. The modified shuttle test (MST) may be more suitable for children with obesity, as it has a much slower start with increments of 0.17 m/s each minute.

In summary, the goals of this pilot study are to outline a varied aerobic exercise program and evaluate it's feasibility and preliminary outcomes, and to assess the feasibility of the modified shuttle test (validity, reliability, repeatability and sensitivity) for evaluating aerobic fitness in pediatric obesity.

## Methods

### Subjects and study design

Pediatric patients with obesity were recruited from the pediatric department of Treatment Center Heideheuvel at Hilversum. Inclusion criteria: children aged 10–18 years with obesity (BMI = 30) who were referred for the multidisciplinary body weight management program. Exclusion criterion: musculo-skeletal disorders. Fifteen consecutive patients met the inclusion criteria. All agreed to participate in the aerobic training study. All children in the aerobic training study were new patients. The local institutional Ethics Committee approved the study protocol. Informed consent was obtained from all participants and their parents.

The baseline measurements were done in the first week of admission. Anthropometric measurements and the cardiopulmonary exercise test were done on day one and day three. On day three and day five the shuttle tests were conducted. The tests were repeated within five days after the training program was finished. The subjects continued their normal daily treatment during the aerobic training study.

### Multidisciplinary body weight management program

The multidisciplinary body weight management program consists of dietary (protein sparing diet), exercise and family-based interventions, medical treatment in case of complications, and behavior modification.

The caloric requirements of each child were measured using the Schofield-formula[44], presuming a P50 weight for length. Thus, caloric restriction is limited as long as the child's weight exceeds this parameter. The guiding principle in the treatment program is that both the child and its parents must learn to accustom themselves to a new dietary pattern (as to content, composition, preparation etcetera) which can also be maintained after the required weight loss has been achieved.

The development of our aerobic exercise program is part of the ongoing development of an effective pediatric body weight management program. The overall goals of the program are loss of fat mass, body weight maintenance and behavior modification.

### Aerobic exercise program

The training group exercised three times a week for 12 weeks[45]. Twice a week in a gymnasium or outdoors and once a week in a swimming pool. The sessions were taken in groups as adolescents feel less inhibited when they exercise in the company of other obese subjects[36]. The standardized training sessions were led by an exercise therapist (physical education teacher). Specific written instructions in the form of a booklet were given to the exercise therapist. Each session lasted 30–60 minutes (mean duration of exercise sessions 43 minutes). We used a structured modular program that enables us to include new patients at any point during the exercise program. In order to increase the physical skills of the obese participants we incorporated a variety of indoor, outdoor and swimming activities in our exercise program.

#### Indoor activities

• Sport games: basketball, handball, korfball (Dutch sports game), field hockey

○ 1 or 2 sets of 3–4 exercises; exercise period 3 to 5 minutes; work-rest ratio between exercises 1:1/2 or 1:1/3 depending on exercise period and level of aerobic fitness; rest between sets 1 to 3 minutes; passive rest recovery (standing or walking at a low pace).

○ sports game: 10 minutes, two teams, adapted rules to ensure an adequate and substantial aerobic challenge.

• Circuits: 2–3 sets; 4–6 exercises; exercise period 1–12 minutes depending on type of circuit; work-rest ratio between exercises 1:1/2 or 1/4 depending on exercise period and/or level of aerobic fitness; rest between sets 1 to 3 minutes; passive rest recovery (standing or walking at a slow pace).

• Running lanes: 2 sets; 5 exercises; exercise period 3–4 minutes; work-rest ratio between exercises 1:1/2 or 1:1/3 depending on exercise period and/or level of aerobic fitness; rest between sets 2 minutes; passive rest recovery (standing or walking at a low pace).

• Tag games: 1–3 games; 1–10 sets depending on number of children; exercise period 3 to 10 minutes; work-rest ratio 1:1/2 or 1:1/3 depending on exercise period; passive rest recovery in which instructions are given how playing the game can be improved or new games are explained.

#### Outdoor activities

• Triathlon: Subsequently exercising on either a normal bicycle or a mountain bike and a kick board followed by swimming. If this was difficult (depending mainly on body mass), the participants use a home trainer (inside) instead of a bicycle/mountain bike and/or walk instead of using the kick board. This differentiation depends mainly on body mass. Exercise period 12 minutes cycle, 12 minutes kick board or walking; no rest between cycling and using a kick board or walking.

Swimming: exercise period 12 minutes; rest between using a kick board or walking and swimming 5 minutes (in which the participants change in to their bathing suits).

#### Swimming activities

• Swim jogging: 1 set; exercise period 30 minutes; every 10 laps (= 150 meters) the participants are informed about their swimming speed; style of swimming is free

• Water polo:

○ 1–3 sets of 3–6 exercises; exercise period 1 minute; work-rest ratio between exercises 1:1/2; rest between sets 2 minutes; passive rest recovery (float in the water or slow swimming).

○ sports game: 12–15 minutes, two teams, adapted rules to ensure an adequate and substantial aerobic challenge.

• Aqua jogging/aqua fitness: 1–2 sets of 4–6 exercises; exercise period 1 minute; work-rest ratio between exercises 1:1/2; rest between sets 2 minutes; passive rest recovery (float in the water or slow swimming).

• Swimming lanes and games: 1–2 sets; 3–4 exercises; exercise period 1–8 minutes; work-rest ratio between exercises 1: 1/4–1/2 depending on exercise period or number of participants; rest between sets 2 minutes; passive rest recovery (float in the water or slow swimming)

During the training sessions, heart rate was measured continuously with a heart rate monitor (Polar Electro Sports Tester S610) and the children were instructed how to use the Polar watches during the exercise sessions: the heart rate had to be at least at their individual training heart rate. The heart rate monitor provided instant feedback (beeping sound) whenever the heart rate became below the target training heart rate, so the children knew when to increase their exercise intensity. During the session, they were continuously encouraged to exercise at their set training heart rate and to increase their speed of movement whenever the heart rate monitor gave a signal. The children were instructed not to increase their movement intensity when the target heart rate was reached. We wanted an indication to which extent the target training heart rates were reached by the participants. Therefore we recorded heart rates in the beginning, in the middle and at the end of the exercise program.

Due to the low self esteem normally seen in the pediatric obesity population[6,46] and to assure successful participation, the training program starts with a training intensity of 50% HR_max_. The activities were designed to keep the level of skill low. In order to induce a training effect, later on in the exercise program we changed over to activities with intensities that were more physiological challenging. This balances physiological training with self-confidence and perceived physical efficacy.

In order to provide an overload, every two weeks the clinical exercise physiologist decided if the exercise intensity could be increased by 5 percent during the next exercise class.

### Outcome measures

#### Body composition (nutritional assessment)

Obesity was defined according to BMI based international cut off points for body mass index[47]. Body weight was measured using a platform beam balance (Seca, Germany) with an accuracy of 0.02 kg. Height was measured with a stadiometer (Holtain, Crymich, UK) with an accuracy of 0.1 cm. Body mass index (BMI) was determined (body weight·height^-2^). Standard deviation scores (z-scores, expressed in sd units) were calculated for BMI (sds-BMI), body weight (sds-body weight) and height (sds-height) from a Dutch reference population [4^th ^Dutch growth study 1997, TNO, The Netherlands].

Fat-free mass (FFM) and fat mass (FM) were determined in the morning after an overnight fast by bioelectrical impedance analysis. Bioelectrical impedance analysis has been shown to be a valid and easy to perform method for the assessment of body composition in pediatric obesity[35,48-50]. Prior to measurements subjects were asked to empty their bladder. Total body resistance was measured by a bioelectrical impedance analyzer (RJL systems, Gouda, The Netherlands, Model BIA 101/S) in supine position. Two electrodes were placed on the dorsal surface of the right hand and foot just proximal to the third metacarpal-phalangeal and metatarsal-phalangeal joints, respectively. Two further electrodes were placed on the dorsal surface of the wrist at the level of the ulnar tubercle, and on the dorsal surface of the right foot between the medial and lateral malleoli.

#### Exercise testing

Aerobic exercise tests were performed on an electronically braked cycle ergometer (Lode Excalibur, Groningen, The Netherlands). All subjects were familiarized with the test and equipment used. Standardized verbal encouragement was given throughout the test to stimulate maximal performance.

Aerobic fitness was assessed by a standard progressive incremental exercise test. Workload was increased by 20 W at 1-minute intervals[51]. The maximal workload (W_max_) was defined as the highest workload maintained during 30 seconds. Continuous respiratory gas analysis and volume measurements, oxygen uptake (VO_2_), and carbon dioxide production (VCO_2_), were performed breath by breath with a triple V valve less mouthpiece and stored in a computerized exercise system (Oxycon Champion, Jaeger, Breda, The Netherlands). Prior to each test, internal gas and volume calibrations were made with certified gases of known standard concentrations. Heart rate was monitored continuously during the tests by 3-leads electrocardiogram (Hewlett-Packard, Amstelveen, The Netherlands). Other measurements taken included ventilation (V_E_) and respiratory exchange ratio (RER= VCO_2_/VO_2_). The highest VO_2 _achieved during the last minute of exercise was taken as VO_2 peak_.

The efficiency of the cardiovascular system during maximal exercise was evaluated using the oxygen pulse (VO_2_/HR). The oxygen pulse is a non-invasive index of the efficiency of the ability of the body to transport oxygen to the working tissue, with more fit subjects having a higher oxygen pulse as compared to less fit subjects.

Efforts were considered to be at a maximum level if at least two out of three of the following criteria were met: 1) cardiac frequency above 195 beats/min[46]; 2) maximal respiratory exchange ratio (i.e. VCO_2_/VO_2 _above 1.0)[52]; 3) subjects showed clinical signs of intense effort and were unable to maintain speed above 50 rpm[53].

Predicted VO_2 peak _(VO_2 peak_%) values were obtained from an age and gender matched Dutch reference population[51].

#### Modified shuttle test

The MST is a standardized 15-level externally paced test for use in adult cystic fibrosis[54]. The test requires the subject to walk up and down a 10-meter course identified by two cones with the walking speed dictated by a prerecorded audio signal. The first speed (0.50 m/s) of walking is referred to as level 1, the second as level 2 and so on. Each level lasts for 1 minute and it continues for 15 levels. The audio set emits a single bleep at regular intervals. The subject should aim to be at the opposite end to the start by the time the bleep sounds. After every minute, the speed of walking is increased by a small increment (0.17 m/s), so the subject walks progressively faster; this is indicated by a triple bleep. Subjects are permitted to run at any time during the test. To help the subjects establish the first very slow speed of walking the operator walks alongside for the first minute.

Heart rate was measured at 5-s intervals using a short-range telemetry device (Polar Electro Sports Tester S610). As with the maximal cycle exercise, standardized verbal encouragement was given throughout the test to stimulate maximal performance. The test was ended when 1) the patient was too tired to maintain the required speed or 2) the patient twice failed to complete a shuttle in the time allowed (more than 0,5 m away from the cone when the bleep sounded). The MST walking distance is the distance walked up and down the 10 m course to the last fully completed cone.

### Data analysis

Data are presented as mean ± SD. Changes within the aerobic exercise group were analyzed with a two-tailed paired t-test. Pearson correlation analyses and linear regression analyses were performed for the modified shuttle test with aerobic indices. Data were analyzed using the Statistical Package for the Social Sciences (SPSS, version 12.0, Chicago. IL, USA).

The construct validity of the MST was measured by relating the instrument to a gold standard: peak oxygen uptake in the progressive incremental exercise test. Correlations between the MST walking distance and aerobic indices were computed.

In order to obtain the test-retest reliability the MST was repeated after two days. The test-retest reliability was studied by computing both the intra-class correlation coefficient (ICC)[52] and the Pearson correlation coefficient between the two assessments.

Pearson correlations between MST and the independent variables were also computed. Stepwise regressions of the significant correlations (p < 0.05) were done to evaluate the contribution of the independent variables to MST walking distance results.

In order to study the sensitivity to change, the MST was used as a functional aerobic outcome measure in the aerobic training study. Sensitivity to change was computed using effect sizes (ES): the observed change divided by the standard deviation of that change[55]. We used Cohen's cut points: ES > 0.2 represents a small change, >0.5 a moderate change, >0.8 is a large change. Effect sizes above 0.5 often indicate clinically relevant changes[56].

## Results

### Training program

The baseline characteristics of the 15 adolescents (mean age: 14.7 ± 2.1 year) who agreed to participate in the aerobic training program are shown in table 1. All subjects were obese (sdsBMI 3.23 ± 0.28)[4].

Since this was a feasibility study, we assured that all adolescents participating in the training program had been exposed to a substantial physical training dose. All 15 participants met the criterion of three times per week and HR equal to or above their individual training HR. In week 1–2, 95% of the heart rates were according to or above the target training heart rate, in week 5–8, 75% and in week 11–12, 82%. The participants enjoyed the program and only one participant missed a training session due to illness.

### Anthropometric characteristics

The baseline and end of training anthropometric characteristics are shown in table 1. Comparing pretraining and end of training period, a significant increase was found for height (0.4%) and significant decreases were found for sds-height (0.3%), body weight (10.6%), sds-body weight (23.9%), BMI (12.6%), sds-BMI (12.5%), total body fat (8.8%) and fat free mass (FFM) (4.8%). All variables showed large changes as indicated by the effect sizes (see table 1)[55].

### Aerobic performance

The changes observed after the 12-week training period for aerobic performance are shown in table 2. Both maximal exercise tests (cycle ergometer and modified shuttle test) were well tolerated by all participants. During the cycle ergometer tests at baseline and at the end of the 12-week training period, mean RER and mean HRmax were respectively, 1.2 ± 0.07, 182.6 ± 7.1 and 1.2 ± 0.06, 185.3 ± 7.1 (table 2). All participants fulfilled at least two criteria for a maximal aerobic exercise test at baseline and at the end of the 12-week training period.

There were significant improvements in VO_2 peak _(mL/min: 17.5%; mL/kg·min: 31.3%; mL/kg·FFM: 23.1%; and %predicted: 16.5%), W_max _(W: 8%; W/kg·FFM: 14.7%), and VO_2 peak_/HR_max _(15.8%). The MST walking distance improved significantly by 32.5%. No changes were found in HR_max_, neither for the cycle ergometer test nor for the modified shuttle.

All aerobic indices showed large to very large relevant changes (see table 2)[55].

### Modified shuttle walk

Significant correlations were found for the MST with aerobic indices: VO_2 peak _(r = 0.79, p < 0.001) and W_max _(r = 0.84, p < 0.001). Simple regression analysis indicated that VO_2 peak _and W_max _accounted for 86% of the variance in the MST walking distance (62% and 71% respectively). None of the anthropometric measures were significantly related to the MST (table 3), despite the moderate correlations of age and height with the MST. The test-retest reliability analysis showed strong intra-class (r = 0.96) and Pearson product moment correlations between the first and second test (r = 0.92). The ICC between peak heart rate from MST and maximal cycle ergometry was r = 0.79. The MST walking distance showed a large relevant clinical change (see table 2)[56].

## Discussion and conclusion

We developed and preliminary evaluated a varied and structured aerobic exercise program for adolescents with severe obesity, and assessed the feasibility of the MST for evaluating aerobic fitness.

The exercise program consists of 12 week, 3 times a week aerobic training with diverse indoor, outdoor and swimming activities. Results from the pilot group of 15 adolescents show a marked improvement in aerobic fitness as measured by peak oxygen uptake, peak work rate, peak oxygen pulse and walking distance. There was also a significant decrease in body weight, sds-body weight, BMI and sds-BMI. The participation rate of this inpatient exercise program was high: 99,8%. The modified shuttle test shows to be a valid, reliable and sensitive outcome.

We showed that our aerobic exercise program effectively improved aerobic performance of adolescents with obesity. Exercise treatments are designed to increase energy expenditure or to increase negative energy balance, although the major impact on energy balance occurs through decreasing caloric intake[7,57]. The effects of exercise can add to dietary interventions to enhance loss of fat mass and improve long-term maintenance i.e. potentiate maintenance of changes in body composition[7]. In addition, aerobic fitness is an important predictor of mortality[6].

In order to induce a training effect, other studies used a training heart rate for the total group, usually HR > 150 beats/min[8,30,32,58,59]. Ebbeling *et al *mention an average HR 60–70% of estimated HR (i.e. 220-age)[3] and no change in training intensity. This does not create an overload during the program, which is important for obtaining a training result[60]. These studies do not take into account the individual differences in maximum HR and rate of improvement of fitness. In the present study exercise intensity was individualized to prevent that some subjects receive insufficient physiological stimulation and others are over stimulated. To optimize results we used individual target training heart rates based on the maximal heart rate obtained during a maximal progressive exercise test. There was a difference between the individual target training HR and the measured HR. We observed that synchronization between starting a new exercise and reaching the higher target training heart rate takes some time. This likely explaines the lower percentage of time of training at the target training heart rate.

Notably, we found a reduction in FFM. The energy deficit caused by the combination of diet and exercise program was very severe: the energy insult produced a 3 kg reduction in FFM despite high loads of exercise. Due to a body weight reduction program FFM is lost, resulting in a decrease in metabolic rate which is a major risk factor for (re)gain of body weight[61]. Physical activity has an anabolic effect on muscle protein metabolism, resulting in an increase in fat-free mass[34,62]. Figueroa-Colon showed a reduction in body weight and body fat after a 10-week intervention with daily aerobic exercise together with a protein-sparing diet but no change in FFM[63]. To seperate the anabolic effect of our exercise program from the catabolic effect of the dietary intervention a randomized controlled study should be conducted with several intervention groups; exercise only, exercise + diet, and diet only. We were not primarily interested in the effects of exercise on β oxidation but in the feasibility of the exercise program and its effects on aerobic fitness. Although longer duration of activities is consistent with the reliance on β oxidation rather than glycolysis as the primary energy source, we think that longer duration of activities is boring for the pediatric population. Therefore, our program has an interval and circuit-like format with relatively short duration of activities. This way we could also keep the participants (who generally have a short attention span) focused on the exercise program.

This study also shows that the MST is a valid, reliable and sensitive measure of aerobic fitness in adolescents with severe obesity. We found strong and highly significant correlations between the distance walked on the MST and aerobic indices. Our results are in agreement with the study of Van Mechelen et al[43]. They validated the maximal multistage 20-meter shuttle run test by Leger et al[42] as an estimate of aerobic fitness and found a correlation of 0.76 between run performance and aerobic fitness in adolescents who are not overweight. We also found a strong positive correlation (r = 0.79) between the MST and VO_2 peak_. In their study as well as in our study a large part of the variation in the MST was explained by VO_2 peak_: 58% and 62% respectively. Although the 20-meter shuttle run test is a valid measure of aerobic fitness in adolescents, the running speed at the beginning of the test is too high and therefore not suitable for adolescents with severe obesity. The modified shuttle test provides an excellent alternative.

One-mile walk/run performance is related to VO_2 peak _in children who are not overweight[64,65]. However, Rowland et al concluded that one-mile run performance may not serve as a strong indicator of cardiovascular fitness[66]. Drinkard *et al*. validated the 12-minute walk/run test as a predictor of peak oxygen uptake in adolescents with obesity[40]. They found a correlation of 0.72 between the 12-minute walk/run distance and VO_2 peak_. Self-paced tests, such as the 6 and 12 minute, and one-mile walk/run have been criticized because they can be influenced by subject motivation[67], contain limited information about physiological changes[68] and are poorly standardized[69]. Lack of standardization in areas know to potentially influence results could impose considerable difficulty in interpreting and comparing walk test results obtained before and after an exercise intervention[69]. The MST is an externally paced maximal incremental field test stressing the patient to an individual symptom limited maximal performance which is independent of body weight. A test that is incremental in nature is advantageous when assessing the outcomes of an exercise program due to the ability to compare pre-training and post-training responses at equivalent exercise intensities[38].

This study has also shown that the MST is suitable for assessing the outcome of interventions aimed at improving aerobic fitness and/or fat mass reduction. Next to the improvement in VO_2 peak _(mL/min) as a result of the aerobic training program, an even larger improvement was found in the distance walked in the MST. This MST improvement was close to the improvement in relative VO_2 peak _(mL/kg·min). This might be explained by the suggestion that obese individuals require a greater portion of their aerobic capacity to move their heavier body mass[70] and since they lost a significant amount of body weight, improvements are probably better reflected in VO_2 peak _per kg body body weight. However, the improvement in absolute VO_2 peak _was also considerable. The ICC between the distance walked on test 1 and 2 was close to 1. In addition, there was no significant difference between HR_max _on test 1 and test 2. These results suggest that a practice walk is not necessary to ensure reliable results in adolescents with severe obesity.

The MST is a suitable test for assessing the physiological capacity in adolescents with obesity, with a greater increase in walking/running speed to stress the more able subjects and because of the slow increase in speed it caters the subjects with less aerobic capacity as well.

These findings have important practical implications regarding the use of the MST as a routine repeatable measure of aerobic fitness in adolescents with obesity. In addition, these results suggest that the MST may be of value in assessing the outcome of exercise interventions with or without body weight reduction.

For reasons of practicability, the MST should be preferred to formal incremental exercise testing. The test can be done in small groups and takes less time to be administered compared to more formal individual exercise tests, which require specialized equipment or expertise. Measuring VO_2 peak _is in our own experience and that of other authors an unpleasant experience for children and adolescents and especially for children and adolescents with obesity. The normal pretest anxiety in measuring peak oxygen consumption may be magnified in this population[58]. As the peak heart rates at the end of the MST and the maximal cycle ergometer test are very similar, the MST peak heart rate can possibly be used to determine the individual target training heart rates. This way, maximal cycle ergometer testing can be omitted without loss of necessary information.

In conclusion, our results show that participation in a varied and structured aerobic exercise program leads to considerable improvements in aerobic performance of adolescents with severe obesity as measured by maximal cycle ergometry (peak oxygen uptake) and the modified shuttle test. We feel that the large improvements in aerobic fitness are not the resultant of diet or growth. A randomized controlled trial is necessary to confirm the value of our exercise program in which, next to aerobic fitness, data on self-efficacy and quality of life, and long-term outcome need to be obtained. The modified shuttle test is an easily administered, reproducible, standardized exercise test for aerobic capacity of adolescents with obesity and easier to administer than a maximal cycle ergometer test. We recommend its use in body weight management programs.

## Abbreviations

BMI body mass index

FFM fat-free mass

FM fat mass

HR heart rate

MST modified shuttle test

RER respiratory exchange ratio

VO_2 peak _peak oxygen uptake

W_max _maximal workload

## Declaration of competing interests

The author(s) declare that they have no competing interests.

## Authors' contributions

PHCK conceived of the study, and participated in its design and coordination, performed the statistical analysis and drafted the manuscript. OvdB-S participated in the coordination and helped to draft the manuscript. HFvS participated in its design and the statistical analysis and helped to draft the manuscript. All authors read and approved the final manuscript.

## Pre-publication history

The pre-publication history for this paper can be accessed here:



## Figures and Tables

**Table 1:** Anthropometric characteristics and effect sizes of the aerobic training group at baseline and at the end of the 12-week training period

	Baseline	End of training	p-value	Effect size
Height cm	167.5 ± 0.07	168.1 ± 0.07	P < 0.001	--
Sds-height	0.20 ± 0.94	0.14 ± 0.94	P = 0.038	--
Body weight kg	103.7 ± 12.4	92.7 ± 11.0	P < 0.001	2.2
Sds-body weight	5.53 ± 1.62	4.21 ± 1.5	P < 0.001	1.3
BMI kg/m^2^	37.4 ± 3.5	32.7 ± 2.7	P < 0.001	1.9
Sds-BMI	3.2 ± 0.28	2.8 ± 0.37	P < 0.001	1.6
Total body fat (FM/TBW%)	43.4 ± 2.8	39.6 ± 2.4	P < 0.001	1.0
FFM	58.7 ± 7.0	55.9 ± 6.7	P = 0.002	1.3

Data are means ± SD. BMI: body mass index; sds: standard deviation score; FM: fat mass; FFM: fat-free mass; TBW: total body weight

**Table 2:** Results and effect sizes for aerobic performance and modified shuttle walk at baseline and at the end of the 12-week training period

		Baseline	12-week training period	p-value	Effect size
**Aerobic performance**					
VO_2 peak_	mL/min	2768.6 ± 416.3	3252.2 ± 490.8	P < 0.0001	2.4
	mL/kg·min	26.8 ± 3.3	35.2 ± 4.5	P < 0.007	2.5
	mL/kg·FFM	47.4 ± 6.4	58.4 ± 7.7	P < 0.0001	2.4
	%predicted	115.5 ± 25.1	134.6 ± 28.0	P < 0.0001	2.7
W_max_	W	200.0 ± 45.4	216 ± 42.2	P < 0.0001	0.8
	W/kg·FFM	3.4 ± 0.6	3.9 ± 0.5	P < 0.0001	1.2
HR_max _cycle ergometer	beats/min	182.6 ± 7.1	185.3 ± 7.1	ns	na
RER		1.2 ± 0.07	1.2 ± 0.06	ns	na
VO_2 peak_/HR_max_	mL/beat	15.2 ± 2.2	17.6 ± 2.7	P < 0.001	1.6
**Modified shuttle walk**					
Distance completed	m	775.0 ± 213.9	1026.7 ± 229.5	P < 0.0001	2.5
HR_max _shuttle	beats/min	187.8 ± 5.0	187.5 ± 4.2	ns	na

VO_2 peak _= peak oxygen uptake; FFM = fat-free mass; W_max _= maximal workload; HRmax = maximum heart rate; RER = respiratory exchange ratio; VO_2 peak_/HR_max _= oxygen pulse. ns = not significant; na = not applicable

**Table 3:** Correlations between baseline modified shuttle walk and anthropometric variables.

	Correlation	p-value
Age	0.34	P = 0.21
Height	0.36	P = 0.19
Sds-height	-0.43	P = 0.17
Body weight	0.18	P = 0.52
Sds-body weight	-0.24	P = 0.46
BMI	-0.10	P = 0.73
Sds-BMI	0.31	P = 0.33
Body fat	0.10	P = 0.73
FM/TBW %	-0.07	P = 0.80
FFM	0.23	P = 0.17

Data are means ± SD. BMI: body mass index; sds: standard deviation score; FM: fat mass; TBW: total body weight.
